# M6AMRFS: Robust Prediction of N6-Methyladenosine Sites With Sequence-Based Features in Multiple Species

**DOI:** 10.3389/fgene.2018.00495

**Published:** 2018-10-25

**Authors:** Xiaoli Qiang, Huangrong Chen, Xiucai Ye, Ran Su, Leyi Wei

**Affiliations:** ^1^Institute of Computing Science and Technology, Guangzhou University, Guangzhou, China; ^2^School of Computer Science and Technology, Tianjin University, Tianjin, China; ^3^Department of Computer Science, University of Tsukuba, Tsukuba, Japan; ^4^School of Software, Tianjin University, Tianjin, China

**Keywords:** N6-methyladenosine site, eXtreme Gradient Boosting, machine learning, feature representation, RNA methylation, feature selection

## Abstract

As one of the well-studied RNA methylation modifications, N6-methyladenosine (m^6^A) plays important roles in various biological progresses, such as RNA splicing and degradation, etc. Identification of m^6^A sites is fundamentally important for better understanding of their functional mechanisms. Recently, machine learning based prediction methods have emerged as an effective approach for fast and accurate identification of m^6^A sites. In this paper, we proposed “M6AMRFS”, a new machine learning based predictor for the identification of m^6^A sites. In this predictor, we exploited a new feature representation algorithm to encode RNA sequences with two feature descriptors (dinucleotide binary encoding and Local position-specific dinucleotide frequency), and used the F-score algorithm combined with SFS (Sequential Forward Search) to enhance the feature representation ability. To predict m^6^A sites, we employed the eXtreme Gradient Boosting (XGBoost) algorithm to build a predictive model. Benchmarking results showed that the proposed predictor is competitive with the state-of-the art predictors. Importantly, robust predictions for multiple species by our predictor demonstrate that our predictive models have strong generalization ability. To the best of our knowledge, M6AMRFS is the first tool that can be used for the identification of m^6^A sites in multiple species. To facilitate the use of our predictor, we have established a user-friendly webserver with the implementation of M6AMRFS, which is currently available in http://server.malab.cn/M6AMRFS/. We anticipate that it will be a useful tool for the relevant research of m^6^A sites.

## Introduction

To date, more than 150 types of RNA modifications have been discovered (Maden, 1990; Wang X. et al., 2014). Of these modifications, N6-methyladenosine (m^6^A) is the most common and abundant one and exists in various species. It is found to be closely associated with diverse biological processes, such as RNA localization and degradation (Wang X. et al., 2014), RNA structural dynamics (Roost et al., 2015), alternative splicing (Liu N. et al., 2015), primary microRNA processing (Alarcón et al., 2015), cell differentiation, and reprogramming (Chen et al., 2015), and regulation of circadian clock (Geula et al., 2015). Thus, identification of m^6^A sites is of great importance for better understanding of their functional mechanisms. In the past few years, high-throughput experimental methods, such as MERIP (Meyer et al., 2012) and m^6^A-seq (Dominissini et al., 2012), have been utilized to identify m^6^A modifications, and more and more m^6^A peaks have been characterized. However, they have the following limitations: (1) they cannot accurately locate the positions of m^6^A sites; (2) they are highly cost; and (3) they are not applicable for the large-scale identification of m^6^A sites. Hence, it is highly desirable to develop fast and accurate computational methods for the identification of m^6^A sites (Chen et al., 2015b, 2016).

In recent years, machine learning based prediction methods have emerged as effective approach for predicting m^6^A sites. For example, Chen et al. (2015a) developed the first machine learning based predictor, called “iRNA-Methyl”, for m^6^A site identification. They exploited physicochemical properties and sequence-order information embedded in PseDNC (pseudo dinucleotide composition) (Liu B. et al., 2015), and used support vector machine for model construction. Later, Liu Z. et al. (2016) proposed to incorporate more additional physicochemical properties coupled with a scalable transformation algorithm into their feature extraction model. To improve the predictive performance, Jia et al. proposed to fuse three types of feature descriptors, such as bi-profile Bayes, dinucleotide composition and KNN scores. Their results showed that this fusion strategy is able to achieve better performance than single one feature descriptor (Jia et al., 2016). Similarly, Xiang et al. (2016b) found that combining binary encoding scheme together with k-mer frequency could contribute to the improved performance. Recently, Zhou et al. (2016) developed “SRAMP”, a powerful prediction tool using multiple types of feature descriptors, including positional binary encoding of nucleotide sequence, k-nearest neighbor encoding, nucleotide pair spectrum encoding, and secondary structure pattern, to train an ensemble predictive model with random forest for the identification of m^6^A sites. SRAMP is reported to achieve relatively good performance as compared to other predictors. More recently, Xiang et al. (2016a) proposed a new predictor called “RNAMethyPre”, using compositional information and position-specific information to build predictive models for the prediction of m^6^A sites on both human and mouse. Additionally, in our previous study, we proposed to use deep learning algorithm to generate high-latent features to improve the predictive performance (Wei et al., 2018d). However, we found that most of existing predictors are species-specific. Currently, there is not any predictor that is capable of predicting m^6^A sites for multiple species.

For this purpose, we proposed a novel sequence-based predictor, namely “M6AMRFS” for detecting m^6^A sites in RNA sequences. For feature extraction (Mrozek et al., 2007, 2013), we proposed a feature representation algorithm to encode sequences with dinucleotide binary encoding and local position-specific dinucleotide frequency. To optimize the feature space, we combined the F-score algorithm with SFS (Sequential Forward Search) (Wei et al., 2018a,c,e) to improve the representation ability of our features. For model training, we trained the optimal feature representations under XGBoost algorithm. Our experimental results showed that the proposed M6AMRFS is able to achieve competitive and robust performance as compared to state-of-the-art predictors for four different species. To the best of our knowledge, this is the first predictor that is applicable for multiple species. Furthermore, we have established a user-friendly webserver that implements the proposed M6AMRFS, which is currently available in http://server.malab.cn/M6AMRFS/. We anticipate that it will be a useful tool complementary for existing tools, facilitating to further reveal the functional mechanisms of m^6^A sites.

## Materials and Methods

### Benchmark Datasets

To predict the m^6^A sites in multiple species, we employed four benchmark datasets from four species, including *Saccharomyces cerevisiae*, *Arabidopsis thaliana*, *Musculus*, and *Homo sapiens*. The detail of the four benchmark datasets is listed in Table 1. For the four benchmark datasets, the positives are the sequences centered with true m^6^A sites, while the negatives are usually the sequences centered with adenines but without any m^6^A peaks detected. The datasets can be found in the following website: http://server.malab.cn/M6AMRFS/.

**Table 1:** Summary of the benchmark datasets from four species.

Datasets	Species	Positives	Negatives	Total	Sequence length	Reference
Dataset-S51	*Saccharomyces cerevisiae*	1307	1307	2614	51 nt	Chen et al., 2015a
Dataset-H41	*Homo sapiens*	1130	1130	2260	41 nt	Chen et al., 2017
Dataset-M41	*Musculus*	725	725	1450	41 nt	Dominissini et al., 2012
Dataset-A101	*Arabidopsis thaliana*	1000	1000	2000	101 nt	Wang and Yan, 2018


### Prediction Framework of the Proposed Predictor

**FIGURE 1:**
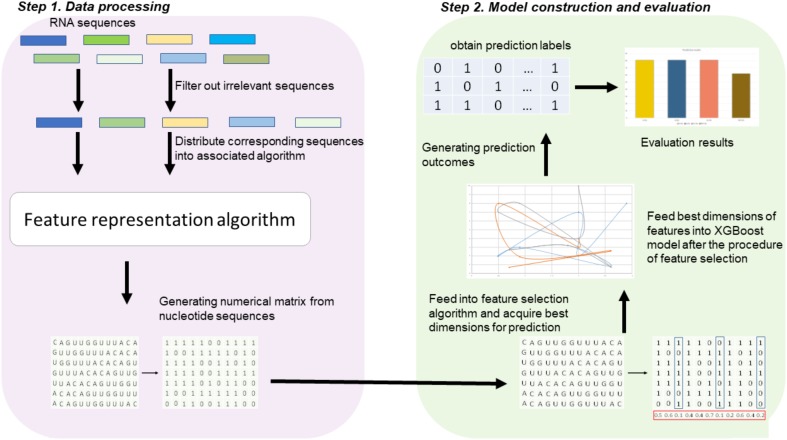
Framework of algorithms proposed in this study. There are two main steps. In the first step, the input RNA sequences are filtered by removing those irrelevant sequences. Then, the remaining sequences are fed into the proposed feature extraction algorithm for feature representation. In the second step, the resulting feature representations are optimized by feature selection, and then, the optimal feature representations are predicted by a XGBoost model.

Figure 1 illustrates the overall procedure of the proposed predictor. As we can see from Figure 1, there are two steps in the predictor. The first step is data pre-processing, including data clean and feature extraction. It filters out those irrelevant sequences from input sequences. Then, the resulting sequences are submitted into the feature representation algorithm, in which the sequences are encoded with feature vectors. The second step is feature optimization and model training. For feature space optimization, we used the F-score algorithm combined with SFS (Sequential Forward Search) to search for the optimal features. Afterward, the resulting optimal feature representations are fed into a well-trained XGBoost model to predict whether the sequences are true m^6^A sites or not. In our predictor, the predicted outcome for each sequence is 0 or 1, where 0 denotes non-m^6^A site and 1 denotes true m^6^A site.

### Feature Representation

In this work, we present a new feature representation algorithm that combines two feature descriptors. One is named “Dinucleotide binary encoding” and the other is “Local position-specific dinucleotide frequency”, which are described as follows,

#### Dinucleotide Binary Encoding

The feature descriptor encapsulates the positional information of the dinucleotide at each position in the sequence. Obviously, there are a total of 16 possible dinucleotides. In this descriptor, each dinucleotide can be encoded into a 4-dimensional 0/1 vector. For example, AA is encoded as (0,0,0,0); AT is encoded as (0,0,0,1); AC is encoded as (0,0,1,0); and so forth, GG is encoded as (1,1,1,1). Therefore, using the dinucleotide binary encoding, we yielded a 160 (=40^∗^4)-dimensional 0/1 vector for the given sequence.

#### Local Position-Specific Dinucleotide Frequency

For a given sequence, the feature vector of this descriptor can be denoted as (*f*_2_, *f*_3_, …, *f*_l_), where *f*_i_ is calculated as follows,

$$f=\frac{1}{\left|N_{i}\right|}C(X_{i-1}X_{i}),2\leq i\leq l$$

where *l* is the length of the given sequence, |*N*_i_| is the length of the *i^th^* prefix string {*X*_1_*X*_2…_*X*_i_} in the sequence, and C (X_i-1_X_i_) is the occurrence number of the dinucleotide X_i-1_X_i_ in position *i* of the *i^th^* prefix string.

#### Feature Selection

**FIGURE 2:**
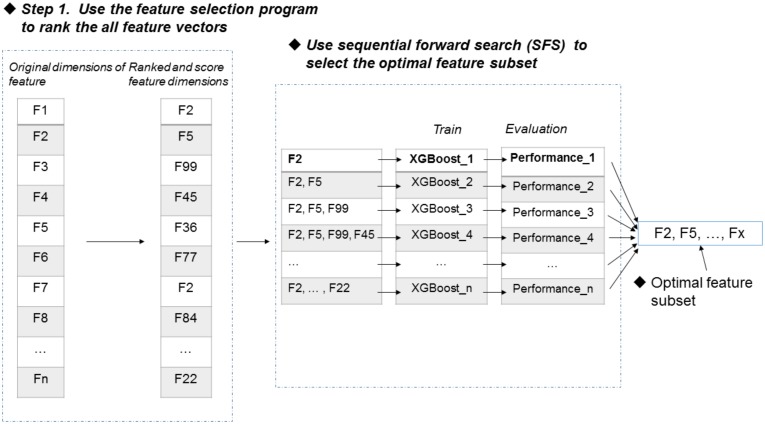
Procedure of feature selection.

Feature selection is an important process to improve the classification performance (Mrozek et al., 2009; Mrozek et al., 2014; Zeng et al., 2016; Zou et al., 2016a,b; Liu, 2017). Here, we used the F-score algorithm together with the SFS strategy to search the most discriminative features (Peng et al., 2005). Figure 2 illustrates the procedure of the feature selection strategy, which is described as follows. Firstly, the F-score algorithm is utilized to rank all the features from the highest scores to the lowest scores, generating a ranked feature list. Secondly, we added the features one by one from the ranked list, and respectively trained the predictive models. Lastly, the feature subset corresponding to the highest accuracy of the predictive model is used as the optimal features. The results of feature selection were discussed in section of “Results and Discussion”.

### XGBoost (eXtreme Gradient Boosting)

eXtreme Gradient Boosting, which was proposed by Chen and Guestrin (2016), has been shown to be a powerful classification algorithm. The general idea of XGBoost is to enumerate several candidates that may be the segmentation points according to the percentile method, and then to find the best segmentation point from the candidates for calculating the segmentation points. The main advantage of XGBoost is to combine multithreading, data compression, and fragmentation methods to improve the efficiency of the algorithm as much as possible. Moreover, the regularization terms added by XGBoost in the loss function can be used to control the complexity of the model and avoid overfitting. Parameters, such as subsamples, max depth, and estimators, are utilized to optimize evaluation performance via parallelization program namely “Grid Search”. For the implementation of XGBoost in our predictor, the range of max depth is set from 2 to 10; learning rate is ranged from 0.1 to 0.8; and estimators are ranged from 1 to 10.

### Performance Evaluation

In this work, four commonly used performance metrics are used for performance evaluation, including Acc (accuracy), Sn (sensitivity), Sp (specificity), and MCC (Mathew’s correlation coefficient), respectively (Zeng et al., 2015; Lai et al., 2017; Zhang et al., 2017; Cheng et al., 2018; Su et al., 2018; Tang et al., 2018; Wei et al., 2018b; Yang et al., 2018). They are formulated as follows

$$\left\{ \begin{array}{c} Sn=\frac{TP}{TP+FN}\times 100\%\\ Sp=\frac{TN}{TN+FP}\times 100\%\\ Acc=\frac{TP+TN}{TP+FN+TN+FP}\times 100\%\\ MCC=\frac{TP\times TN-FP\times FN}{\sqrt{(TP+FN)(TN+FP)(TP+FP)\left(TN+FN\right)}} \end{array}\right.$$

where TP denotes true positive; TN denotes true negative; FP denotes false positive; and FN denotes false negative. Sn measures the predictive ability of a predictor for positive samples while Sp measures the predictive ability of a predictor for negative samples. Acc and MCC are two metrics measuring the overall performance of a predictor.

Besides, we used Receiver Operating Characteristic (ROC) curve to intuitively evaluate the overall performance (Liu et al., 2013, 2016b). It is plotted with true positive rate (TPR) against false positive rate (FPR) under different classification thresholds. The TPR is the same with sensitivity as described above, while FPR is calculated as 1-specificity. Area under ROC curve (AUC) is usually used as an evaluation metric (Liu et al., 2016a, 2017). The value of AUC ranges from 0.5 to 1. If the AUC is close to 1, it indicates that the predictor has excellent performance. If the AUC approaches to 0.5, the predictor does not perform well for prediction.

Additionally, we used 10-fold cross validation method and jackknife test to evaluate the predictive performance (Wei et al., 2017a; Zeng et al., 2017a,b; Liao et al., 2018; Zou et al., 2018). The two evaluation methods were chosen since existing methods in the literature used them for performance evaluation.

## Results and Discussion

### Comparison of XGBoost and Other Classifiers

**FIGURE 3:**
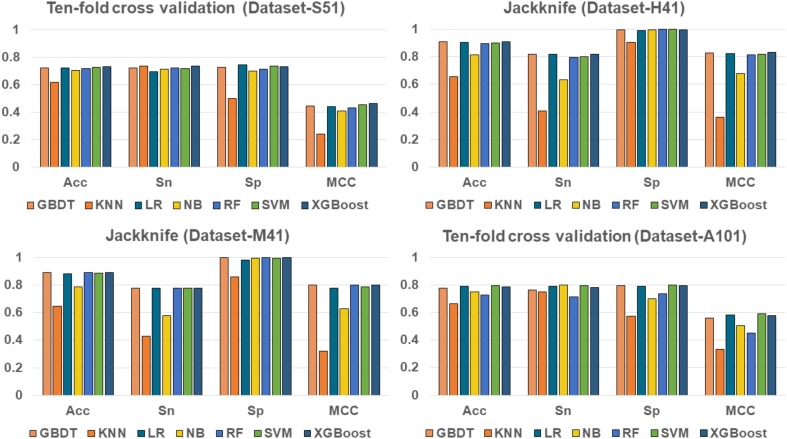
Performance of XGBoost and other classifiers on four benchmark datasets.

To evaluate the effectiveness of the XGBoost classifier, we compared it with five commonly used machine learning algorithms, including Random Forest (RF) (Liu B. et al., 2015; Li et al., 2016; Wei et al., 2017b), Naïve Bayes (NB), Logistic Regression (LR), K-Nearest Neighbors (KNN)(Huang and Li, 2018), Support Vector Machine (SVM) (Song et al., 2010, 2012, 2018; Wang M. et al., 2014; Wei et al., 2017), and Gradient Boosting Decision Tree (GBDT) (Liao et al., 2018), respectively. For fair comparison, the machine learning algorithms were trained and evaluated with 10-fold cross validation on the benchmark datasets, respectively. The performance of different classifiers is illustrated in Figure 3. The detailed results are presented in Table 2.

**Table 2:** Performances of XGBoost and other machine learning algorithms.

Dataset-S51	Acc	Sn	Sp	MCC	Dataset-H41	Acc	Sn	Sp	MCC
GBDT	0.7234	0.7200	0.7269	0.4468	GBDT	0.9089	0.8204	0.9973	0.8308
KNN	0.6167	0.7337	0.4996	0.2400	KNN	0.6566	0.4062	0.9071	0.3620
LR	0.7192	0.6924	0.7460	0.4390	LR	0.9066	0.8204	0.9929	0.8257
NB	0.7050	0.7100	0.7001	0.4101	NB	0.8155	0.6327	0.9982	0.6779
RF	0.7165	0.7192	0.7138	0.4331	RF	0.8982	0.7965	1.0000	0.8135
SVM	0.7257	0.7169	0.7345	0.4515	SVM	0.9018	0.8035	1.0000	0.8195
XGBoost	0.7314	0.7345	0.7284	0.4629	XGBoost	0.9089	0.8195	0.9982	0.8311
**Dataset-M41**	**Acc**	**Sn**	**Sp**	**MCC**	**Dataset-A101**	**Acc**	**Sn**	**Sp**	**MCC**
GBDT	0.8890	0.7779	1.0000	0.7979	GBDT	0.7795	0.7624	0.7967	0.5594
KNN	0.6448	0.4303	0.8593	0.3207	KNN	0.6638	0.7524	0.5752	0.3329
LR	0.8807	0.7793	0.9821	0.7775	LR	0.7914	0.7910	0.7919	0.5829
NB	0.7862	0.5793	0.9931	0.6288	NB	0.7517	0.8005	0.7029	0.5057
RF	0.8890	0.7779	1.0000	0.7979	RF	0.7260	0.7152	0.7367	0.4520
SVM	0.8848	0.7766	0.9931	0.7884	SVM	0.7971	0.7957	0.7986	0.5943
XGBoost	0.8890	0.7779	1.0000	0.7979	XGBoost	0.7890	0.7824	0.7957	0.5781


As shown in Table 2 and Figure 3, XGBoost outperforms the other classifiers on three out of the four datasets, with the exception of Dataset-A101, for which the SVM classifier is slightly better than the XGBoost, which is the second best among the compared classifiers. For those datasets that the XGBoost outperforms other classifiers, the XGBoost is able to achieve higher Acc and MCC. To be specific, our Acc and MCC are 0.7314 and 0.4629 in the Dataset-S51, 0.6 and 1.1% higher than that of the runner-up SVM. Similar results are observed in the Dataset-H41; XGBoost leads by 0.71 and 1.2% in terms of Acc and MCC, respectively. Moreover, in the Dataset-M41, the performances of our XGBoost are the same with the RF and GBDT in terms of Acc, Sn, Sp, and MCC, respectively. In summary, our results demonstrate that as compared to other commonly used classifiers, the XGBoost shows generally better and more robust performance to classify true m^6^A sites to non- m^6^A sites from different species.

### Impact of Feature Selection

**FIGURE 4:**
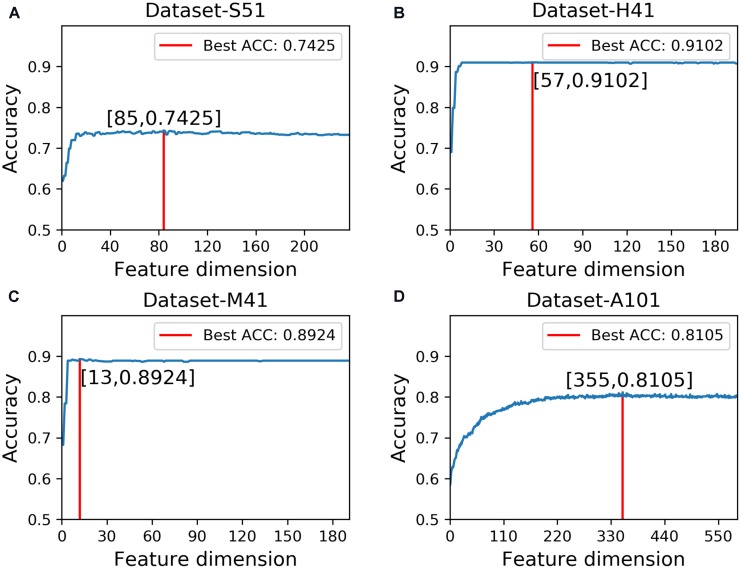
Results of feature selection via varying the feature number. **(A)** denotes the results of feature selection on Dataset-S51. **(B)** denotes the results of feature selection on Dataset-H41. **(C)** denotes the results of feature selection on Dataset-M41. **(D)** denotes the results of feature selection on Dataset-A101.

In this study, we employed the F-score with the SFS for feature selection. The results of feature selection are summarized in Table 3 and illustrated in Figure 4 as well. As seen from Table 3, before feature selection, the performances of the predictive model in the Dataset-S51 are 0.7314, 0.7345, 0.7284, and 0.4629 in terms of Acc, Sn, Sp, and MCC, respectively. After applying the feature selection, we observed that the performances in terms of all the metrics were improved. To be specific, the Acc and MCC were improved to 0.7425 and 0.4852, respectively. This indicates that the feature selection strategy to yield more informative features to distinguish true m^6^A sites from non-m^6^A sites. For the other datasets from different species, similar results were observed. We can see from Table 3 that almost all the performances were improved by using feature selection, demonstrating that feature selection is an effective way to enhance the predictive performance of the predictor. Moreover, Figure 4 illustrates the Acc of the features by varying the feature number when conducting feature selection. As seen in Figure 4, we pointed out the optimal feature number and their corresponding highest Acc for each dataset. The optimal feature number for the four datasets are 85, 57, 13, and 355, giving the highest Acc of 0.7425, 0.9102, 0.8924, and 0.8105, respectively.

**Table 3:** Performance of features before and after feature selection.

Datasets	Methods	Acc	Sn	Sp	MCC
*Dataset-S51*	*Before*	*0.7314*	*0.7345*	*0.7284*	*0.4629*
	*After*	*0.7425*	*0.7521*	*0.7330*	*0.4852*
*Dataset-H41*	*Before*	*0.9089*	*0.8195*	*0.9982*	*0.8311*
	*After*	*0.9102*	*0.8204*	*1.0000*	*0.8339*
*Dataset-M41*	*Before*	*0.8890*	*0.7779*	*1.0000*	*0.7979*
	*After*	*0.8924*	*0.7890*	*0.9959*	*0.8022*
*Dataset-A101*	*Before*	*0.7890*	*0.7824*	*0.7957*	*0.5781*
	*After*	*0.8105*	*0.8067*	*0.8143*	*0.6210*


### Comparison With Other Feature Representation Algorithms

To examine the performance of the proposed feature algorithm, we evaluated and compared it with existing feature representation algorithms, including RFH, PseDNC, PCP (physical and chemical properties), KNN (K-Nearest Neighbors), and AthMethPre, respectively. These algorithms were reported to have relatively strong power for the identification of m^6^A sites. Thus, they were chosen for comparison. The results of the above algorithms were presented in Table 4. As we can see from Table 4, the proposed features are competitive with the best-performing AthMethPre other feature representation methods and remarkably outperform the other existing features in all the four datasets. Note that for the Dataset-S51 and the Dataset-A101, our method performs slightly worse than the best-performing AthMethPre; while for the other two datasets, our method is slightly better. As well known, for the genome-wide identification, the running time for a predictor is important as well. Therefore, we further compared the feature number of AthMethPre and our feature representation method. We found that the feature number of the AthMethPre method for each dataset are 540, 500, 500, and 740, while ours are 85, 57, 13, and 355, respectively. As can be seen, our feature numbers for all the four datasets are averagely much fewer than the AthMethPre method. This indicates that the computation time by our predictive models costs less. In general, it can be concluded that our features are at least effective for the representatives of m^6^A sites in multiple species with different sequence lengths.

**Table 4:** Comparison with other feature representation algorithms.

Dataset-S51	Acc	Sn	Sp	MCC	Dataset-H41	Acc	Sn	Sp	MCC
RFH	0.7295	0.7582	0.7008	0.4598	RFH	0.9097	0.8195	1	0.8332
PseDNC	0.64	0.6993	0.5807	0.282	PseDNC	0.6956	0.5973	0.7938	0.3989
PCP	0.627	0.6389	0.6151	0.2541	PCP	0.6447	0.6177	0.6717	0.2898
KNN	0.7131	0.6917	0.7345	0.4266	KNN	0.8235	0.7363	0.9106	0.657
AthMethPre	0.7536	0.7605	0.7467	0.5073	AthMethPre	0.9071	0.8142	1	0.8286
Our features	0.7425	0.7521	0.733	0.4852	Our features	0.9102	0.8204	1	0.8339
**Dataset-M41**	**Acc**	**Sn**	**Sp**	**MCC**	**Dataset-A101**	**Acc**	**Sn**	**Sp**	**MCC**
RFH	0.8903	0.7848	0.9959	0.7987	RFH	0.7993	0.7705	0.8281	0.5996
PseDNC	0.6228	0.6386	0.6069	0.2456	PseDNC	0.8138	0.8057	0.8219	0.6277
PCP	0.6166	0.5669	0.6662	0.2343	PCP	0.8257	0.8281	0.8233	0.6514
KNN	0.8283	0.7448	0.9117	0.6659	KNN	0.8238	0.8462	0.8014	0.6483
AthMethPre	0.8897	0.7793	1	0.799	AthMethPre	0.85	0.85	0.85	0.7
Our features	0.8924	0.789	0.9959	0.8022	Our features	0.8105	0.8067	0.8143	0.6210


### Comparison With State-of-the-Art Predictors

To assess the effectiveness of our predictor, we compared it with existing predictors including pRNAm-PC (Liu Z. et al., 2016), MehtyRNA (Chen et al., 2017), and RFAthM6A (Wang and Yan, 2018), respectively. There were chosen since they were reported to have the best performance on the four benchmark datasets used in this work. The results were presented in Table 5.

**Table 5:** Results of the proposed predictor and the state-of-the-art predictors on benchmark datasets from different species.

Dataset-S51	Acc	Sn	Sp	MCC	Dataset-H41	Acc	Sn	Sp	MCC
pRNAm-PC	0.6974	0.6972	0.6975	0.4000	MethyRNA	0.9038	0.8168	0.9911	N.A.
M6AMRFS	0.7425	0.7521	0.7330	0.4852	M6AMRFS	0.9102	0.8204	1.0000	0.8339
**Dataset-M41**	**Acc**	**Sn**	**Sp**	**MCC**	**Dataset-A101**	**Acc**	**Sn**	**Sp**	**MCC**
MethyRNA	0.8839	0.7779	1.0000	N.A.	RFAthM6A	0.8545	0.8738	0.8352	0.7095
M6AMRFS	0.7933	0.8281	0.7584	0.588	M6AMRFS	0.8105	0.8067	0.8143	0.6210


*N.A., denotes not available.*

As shown in Table 5, M6AMRFS outperforms pRNAm-PC on the Dataset-S51. The Acc, Sn, Sp, and MCC by our predictor are 0.7425, 0.7521, 0.7339, and 0.4852, respectively. The performances are higher than that of the second best pRNAm-PC on this dataset. To be specific, our overall performances are 0.0451 and 0.0852 higher in terms of Acc and MCC, respectively. As for the other datasets (Dataset-H41 and Dataset-M41), we observed similar results that our overall performance outperforms the existing predictors. Only on Dataset-A101, our predictor performs slightly worse than RFAthM6A. To be concluded, our results demonstrate that the proposed predictor is better than existing predictors or at least competitive with existing predictors on multiple benchmark datasets from different species. Importantly, our predictor exhibits robust performance for multiple species, demonstrating that our predictor is able of capturing the characteristics of m^6^A sites in different species. This also implies that the m^6^A sites from different species might share the common patterns.

## Conclusion

In this study, we have developed a machine learning based predictor, namely M6AMRFS, for the identification of m^6^A sites in multiple species. We have conducted a series of comparative study, and our experimental results indicate that our predictor is at least competitive as compared to previously published predictors. Importantly, we found that our predictor is able to achieve robust performance in several species. To the best of our knowledge, it is the first predictor that can provide predictions in multiple species. For further analysis, we found that the robust performance contributes to the following two possible reasons. One reason is the XGBoost classifier we used for model training. We have compared XGBoost with other machine learning algorithms. XGBoost is shown to perform better than other classification algorithms. The other reason is that our feature selection strategy helps to adaptively select the optimal features for specific species. We anticipate that the tool and webserver we have established will be useful for facilitating to reveal the functional mechanisms of m^6^A sites.

## Author Contributions

XQ and HC wrote the manuscript. HC developed the webserver and analyzed the results. XY analyzed the results. RS and LW designed the experiments. All authors read and approved the manuscript.

## Conflict of Interest Statement

The authors declare that the research was conducted in the absence of any commercial or financial relationships that could be construed as a potential conflict of interest.
